# Long-term effects on carotid intima-media thickness after radiotherapy in patients with nasopharyngeal carcinoma

**DOI:** 10.1186/1748-717X-8-261

**Published:** 2013-11-07

**Authors:** Tai-Lin Huang, Hsuan-Chih Hsu, Hui-Chun Chen, Hsin-Ching Lin, Chih-Yen Chien, Fu-Min Fang, Chih-Cheng Huang, Hsueh-Wen Chang, Wen-Neng Chang, Chi-Ren Huang, Nai-Wen Tsai, Chia-Te Kung, Hung-Chen Wang, Wei-Che Lin, Ben-Chung Cheng, Yu-Jih Su, Ya-Ting Chang, Chuang-Rung Chang, Teng-Yeow Tan, Cheng-Hsien Lu

**Affiliations:** 1 Departments of Hematology-Oncology, Chang Gung University College of Medicine, Kaohsiung, Taiwan; 2 Institute of Biotechnology and Department of Medical Science, National Tsing Hua University, Hsinchu, Taiwan; 3 Departments of Radiation Oncology, Chang Gung University College of Medicine, Kaohsiung, Taiwan; 4 Department of Otorhinolaryngology, Chang Gung University College of Medicine, Kaohsiung, Taiwan; 5 Department of Neurology, Chang Gung University College of Medicine, Kaohsiung, Taiwan; 6 Department of Biological Science, National Sun Yat-Sen University, Kaohsiung, Taiwan; 7 Department of Emergency Medicine, National Sun Yat-Sen University, Kaohsiung, Taiwan; 8 Department of Neurosurgery, National Sun Yat-Sen University, Kaohsiung, Taiwan; 9 Department of Radiology, National Sun Yat-Sen University, Kaohsiung, Taiwan; 10 Medicine, Kaohsiung Chang Gung Memorial Hospital, Chang Gung University College of Medicine, Kaohsiung, Taiwan

**Keywords:** Atherosclerosis, Nasopharyngeal carcinoma, Radiotherapy, Risk factors

## Abstract

**Background:**

Vascular abnormalities are the predominant histologic changes associated with radiation in nasopharyngeal carcinoma (NPC). This study examined if the duration after radiotherapy correlates with the progression of carotid intima-media thickness (IMT) and investigated its relationship with inflammatory markers.

**Methods:**

One hundred and five NPC patients post-radiotherapy for more than one year and 25 healthy control subjects were examined by B-mode ultrasound for IMT measurement at the far wall of the common carotid artery (CCA). Surrogate markers including lipid profile, HbA1c, and high sensitive C-reactive protein (hs-CRP) were assessed.

**Results:**

The IMT of CCA was significantly increased in NPC patients and carotid plaque was detected in 38 NPC patients (38/105, 36.2%). Significant risk factors for carotid plaques included age, duration after radiotherapy, and HbA1c levels. Age, duration after radiotherapy, *hs*-CRP, HbA1c, and platelet count positively correlated with IMT. The cut-off value of age and duration after radiotherapy for the presence of plaque was 52.5 years and 42.5 months, respectively. In NPC subjects, multiple linear regression analysis revealed that age, gender, duration after radiotherapy and platelet counts were independently associated with CCA IMT. After adjustments for age, gender and platelet counts, IMT increased in a linear manner with duration after radiotherapy.

**Conclusions:**

Radiation-induced vasculopathy is a dynamic and progressive process due to late radiation effects. Extra-cranial color-coded duplex sonography can be part of routine follow-up in NPC patients aged ≥50 years at 40 months post-radiotherapy.

## Background

Nasopharyngeal carcinoma (NPC) is a rare disease in most parts of the world, with an annual crude incidence rate of 1.1 per 100 000 [1]. However, it is one of the leading causes of cancer among the Chinese, particularly those from southeastern China and Taiwan, where the incidence is 20–40 per 100,000 person-years [1]. With ongoing improvements in cancer therapy and healthcare, cancer can be considered a chronic disease for some patients [2]. Treatment of combined chemotherapy and radiotherapy prolongs survival in patients with head and neck cancers [1].

Abundant circumstantial evidence indicates that oxidative stress and inflammation may be involved in the development of radiation-induced chronic tissue damage in a dynamic and progressive process without a clear cut-off time point that delineates the acute from the late radiation effects [3]. Exposure to ionizing radiation leads to the increased generation of reactive oxygen species and free radicals. When cellular repair and free radical scavenger systems unable to counteract these free radical insults, oxidative damage occurs and affects cellular structure and function, and the activation of early response transcription factors and signal transduction pathways [3].

In clinical practice, vascular abnormalities are the predominant histologic changes seen in irradiation of head and neck cancers and these are observed more than 6 months post-irradiation [4]. Several articles report variables on radiation-related extra-cranial vasculopathy after radiotherapy for NPC but most of these are retrospective studies with variable follow-up periods or the same study group [5-9]. Carotid intima media thickness (IMT) is a good indicator of the severity of atherosclerotic disease [10,11]. To date, little is known about the long-term effects of carotid IMT changes in NPC patients following radiotherapy. This study tested the hypothesis that the duration of radiotherapy correlates with the progression of carotid IMT and is related to oxidative stress and inflammation. The successful translation of these approaches to the clinics offers the promise of improving not only long-term survival but also the quality of life for radiotherapy patients.

## Patients and methods

### Study design

This is a single-center, prospective, case–control study conducted at Chang Gung Memorial Hospital-Kaohsiung, a medical center and main referral hospital serving an area of 3 million inhabitants in southern Taiwan.

### Inclusion and exclusion criteria

From August 2010 to May 2011, 113 diagnosed NPC patients who were one year post-radiotherapy were evaluated. The hospital’s Ethics Committee approved the study protocol and all of the enrolled patients or their relatives provided written informed consent. Patients were excluded if: 1) the duration after radiotherapy was less than one year; 2) there were evidences of fever or a history of infection one week before the study; 3) history of chemotherapy for relapse of cancer three months prior to the study; 4) history of cerebral infarctions, coronary artery disease status post-percutaneous trans-luminal coronary angioplasty or bypass surgery, or renal failure requiring hemodialysis or peritoneal dialysis before the treatment of NPC, and 5) ultrasound for IMT could not be measured under the following circumstances including severe fibrosis, heavy calcification and multiple plaques. Eight patients, including five with unilateral and/or bilateral internal carotid artery (ICA) severe stenosis (>75%) and/or occlusion, and three with severe fibrosis were excluded. Thus, only 105 cases were enrolled in this study.

### Diagnostic criteria and therapeutic regimens of nasopharyngeal carcinoma

Experienced pathologists diagnosed NPC histologically while multi-disciplinary teams administered treatment for all patients. The therapeutic protocol was according to the NCCN (National Comprehensive Cancer Networks) Clinical Practice Guidelines in Oncology-Head and Neck cancers (USA) and the Kaohsiung CGMH Head and Neck Oncology Group, Chang Gung Memorial Hospital Cancer Center. The therapeutic strategies were as follows: radiotherapy alone for patients with stages I-IIA (American Joint Committee on Cancer 2010 (AJCC)) and concurrent chemo-radiation (CCRT) for those with stages IIB-IV. According to the staging system of AJCC for NPC, the distribution of the 105 patients was as follows: two Stage I, three StageIIa, 41 Stage IIb, 42 StageIII, and 17 Stage IV. The technical details of radiotherapy for NPC have been described previously [12]. The prescribed dose ranged from 68.4 to 75.6 Gy depending on the tumor stage, with a daily fraction of 1.8 Gy and 5 fractions per week. The prescribed first neo-adjuvant chemotherapy regimen was 70 to 80 mg/m^2^ cisplatin intravenous bolus on day 1 and 700 to 800 mg/m^2^/day continuous intravenous infusion of fluorouracil on days 1 to 4, and CCRT chemotherapy regimen were weekly Cisplatin 40 mg/m^2^ IV infusion for five cycles.

### Clinical assessment

All patients received complete medical and neurologic examinations, as well as extra-cranial color-coded duplex sonography (ECCS). From a review of the clinical records of the NPC patients, body mass index (BMI) was calculated and the age of onset and follow-up period after radiotherapy was registered. The methodology of CCA IMT measurement is based on recent studies [13].

### Assessment of atherosclerosis

Images were obtained from a B-mode ultrasound system (Philips HDI 5000 System, ATL-Philips, Bothell, WA) equipped with a 4–10 MHz linear array transducer. Subjects were examined according to a standardized protocol by an experienced ultrasound technologist who was blind to the clinical history of the subjects. Both the left and right CCA were routinely scanned and defined as the 1-cm vascular wall segment of the carotid artery immediately proximal to the dilatation of the bifurcation plane. The images were optimized so that only the far wall was visualized in a single longitudinal view.

All IMT measurements were made at the smooth plaque-free portion on the far wall of the CCA in the longitudinal plane along a 1-cm length proximal to the carotid bulb. The IMT was defined as the distance between the intima-blood interface and the adventitia-media junction. Plaque was defined as a localized wall thickening at least twice the thickness of the adjacent IMT. The obtained images were transferred to a workstation and the IMT was automatically measured using a computer software program (Q-LAB, ATL-Philips). These measurements were again performed in a single-blind fashion and mean CCA IMT was the average of measurements obtained.

### Biochemical analysis

Blood samples were obtained by antecubital vein puncture in a fasting, non-sedative state between 09:00 and 10:00 AM in the control and study groups to exclude the possible influence of circadian variations. The samples were analyzed by the central laboratory of Chang Gung Memorial Hospital-Kaohsiung for serum levels of triglycerides, total cholesterol, high-density lipoprotein cholesterol (HDL-C), low-density lipoprotein cholesterol (LDL-C), blood sugar, HBA1c, and high sensitive C-reactive protein (hs-CRP).

### Statistical analysis

Four separate series of statistical analyses were performed using the SAS software package, version 9.1 (2002, SAS Statistical Institute, Cary, North Carolina). Data were expressed as mean ± SD or median (inter-quartile range). Categorical variables were compared using the Chi-square test or Fisher’s exact test. Continuous variables were compared using the Student’s t-test. Those that were not normally distributed were logarithmically transformed to improve normality and then compared. First, the demographic data between NPC patients and healthy controls were compared.

Second, the risk factors of carotid plaques in NPC patients were evaluated. The receiver operating characteristic (ROC) curves was generated for age and duration after radiotherapy and the areas under the ROC curves (AUC) were calculated for each parameter and compared. Third, correlation analysis was used to evaluate the relationship between mean IMT and variables that included duration of radiotherapy, age, gender, or cholesterol-profile, and hs-CRP concentration. For this purpose, the duration of radiotherapy in the control group was defined as 0. Fourth, two stepwise models of multiple linear regression analysis were performed to assess the impact of independent variables on mean IMT. The assessment of factors that significantly correlated with mean IMT were carried out initially by model 1 multiple linear regression analysis, while the identified significant factors were further subjected to model 2 multiple linear regression analysis.

## Results

### Baseline characteristics of the study patients

The baseline characteristics and laboratory data of the 105 adult NPC cases and 25 healthy volunteers were listed in Table 1. The characteristics of the two groups were similar as regards age, gender, and body mass index. There were no significant differences between the two groups in terms of white blood cell (WBC) counts, platelet counts, HDL, Glucose and glycosylated hemoglobin (HbA1c). However, serum total cholesterol, LDL, triglyceride and *hs*-CRP level were significantly higher in NPC patients than in control subjects (*p* < 0.05). The RBC counts were significantly lower in NPC patients than in control subjects (*p* < 0.05). Furthermore, the IMT of CCA on either side or the mean CCA IMT was significantly increased in NPC patients (left CCA IMT, *p* < 0.001; right CCA IMT, *p* < 0.001; and CCA IMT (average), *p* < 0.001).

**Table 1:** Demographic data and vascular risk factors in nasopharyngeal carcinoma patients and normal controls

	***NPC Patients (n = 105)***	***Controls (n = 25)***	***p value***
Age, y	*52.43 ± 10.23*	*50.68 ± 11.49*	*0.280*
*Gender (female; male)*	*33; 72*	*9; 16*	*0.660*
*Body mass index, kg/m*^*2*^	*23.78 ± 3.61*	*22.61 ± 2.95*	*0.142*
*Median (IQR)duration after radiotherapy (months)*	*48 (31, 77)*	*---*	
*Right CCA IMT, mm*	*1.025 ± 0.611*	*0.612 ± 0.074*	*<0.001*
*Left CCA IMT, mm*	*1.036 ± 0.541*	*0.643 ± 0.111*	*<0.001*
*Mean CCA IMT, mm*	*1.031 ± 0.539*	*0.634 ± 0.111*	*<0.001*
*Cholesterol, mg/dL*			
*Total*	*201.96 ± 37.52*	*187.17 ± 24.28*	*0.021*
*HDL-C*	*61.07 ± 15.23*	*67.62 ± 14.55*	*0.059*
*LDL-C*	*118.22 ± 35.06*	*102.13 ± 18.39*	*0.003*
*Triglyceride, mg/dL*	*114.39 ± 63.82*	*86.83 ± 35.74*	*0.006*
*Glucose, mg/dL*	*90.96 ±14.03*	*86.79 ± 8.12*	*0.165*
*HBA1c*	*5.70 ± 0.47*	*5.56 ± 0.23*	*0.146*
*hs-CRP, mg/L*	*3.50 ± 1.38*	*1.12 ± 1.00*	*<0.001*
*WBC counts (X10*^*3*^*/mL)*	*4.86 ± 1.62*	*5.32 ± 1.02*	*0.189*
*RBC counts (X10*^*6*^*/ml)*	*4.61 ± 0.67*	*4.95 ± 0.49*	*0.035*
*Platelet counts (X10*^*3*^*/mL)*	*225.74 ± 64.74*	*207.75 ± 39.44*	*0.195*

Values are expressed in mean ± SD unless otherwise indicated.

Abbreviations: IQR inter-quartile range, HBA1c glycosylated hemoglobin.

### Carotid plaques and vascular risk factors

Carotid plaque was detected in 38 NPC patients (38/105, 36.2%). The locations of CCA involved included 3 on the left, 3 on the right, and 32 bilateral. Significant risk factors for carotid plaques included age (*p* < 0.001), duration after radiotherapy (*p* = 0.023), HbA1c (P = 0.022), and CD62P level (P = 0.049) (Table 2). To demonstrate the relationship of age and duration after radiotherapy with the presence of plaque in NPC patients, the ROC curves were generated for both age and duration after radiotherapy. The AUC for age and duration after radiotherapy were 0.770 (*p* < 0.001, 95% CI = 0.679-0.860) and 0.624 (*p* = 0.036, 95% CI = 0.508-0.741), respectively. The cut-off values of age and duration after radiotherapy for presence of carotid plaque were 52.5 years old (sensitivity 84.2% and specificity 66%) and 42.5 months (sensitivity 68.4% and specificity 50%), respectively.

**Table 2:** Vascular risk factors for carotid plaques in nasopharyngeal carcinoma patients

	***Without carotid plaques (n = 67)***	***With carotid plaques (n = 38)***	***p value***
*Age, y*	*48.86 ± 9.8*	*58.79 ±7.77*	*<0.001*
*Gender (female; male)*	*23; 44*	*10; 28*	*0.395*
*Body mass index, kg/m*^*2*^	*23.65 ±3.62*	*24.01 ± 3.63*	*0.624*
*Median (IQR) duration after radiotherapy (months)*	*45.0 (25.5, 63.5)*	*63.5 (32.5, 9.5)*	*0.023*
*Cholesterol, mg/dL*			
*Total*	*200.39 ± 32.26*	*204.67 ± 45.58*	*0.617*
*HDL-C*	*60.78 ± 16.26*	*61.59 ± 13.44*	*0.797*
*LDL-C*	*116.39 ± 29.58*	*121.37 ± 43.20*	*0.536*
*Triglyceride, mg/dL*	*120.96 ± 71.34*	*103.01 ± 46.89*	*0.174*
*Glucose, mg/dL*	*89.18 ± 9.77*	*94.02 ± 19.08*	*0.157*
*HBA1c*	*5.60 ± 0.29*	*5.86 ± 0.64*	*0.022*
*hs-CRP, mg/L*	*3.81 ± 1.81*	*2.96 ± 1.03*	*0.406*
*WBC counts (X10*^*3*^*/mL)*	*4.83 ± 1.74*	*4.91 ± 1.42*	*0.814*
*RBC counts (X10*^*6*^*/ml)*	*4.51 ± 0.59*	*4.78 ± 0.76*	*0.052*
*Platelet counts (X10*^*3*^*/mL)*	*221.06 ± 65.66*	*233.83 ± 63.17*	*0.342*

Values are expressed in mean ± SD unless otherwise indicated.

Abbreviations: IQR inter-quartile range, HBA1c glycosylated hemoglobin.

### Correlation analysis of the effects of vascular risk factors on CCA IMT

Correlation analysis was used to test vascular risk factors on CCA IMT (average). The correlation coefficients and *p* values were as follows: age (r = 0.598, *p* < 0.001), duration after radiotherapy (months) (r = 0.615, *p* < 0.001), *hs*-CRP (r = 0.222, *p* = 0.014), HbA1c (r = 0.200, *p* = 0.049), and platelet count (r = 0.326, *p* < 0.001) (Table 3).

**Table 3:** Correlation analysis of the effects of candidate risk factors on CCA IMT in NPC patients

***Variables***	***Correlation coefficient***	***P value***
*Age*	*0.598*	*<0.001*
*Duration of radiotherapy therapy (months)*	*0.615*	*<0.001*
*hs-CRP*	*0.222*	*0.014*
*Glucose, mg/dL*	*0.147*	*0.144*
*HBA1c*	*0.200*	*0.049*
*Total Cholesterol*	*-0.074*	*0.459*
*HDL*	*-0.030*	*0.765*
*LDL-C*	*0.013*	*0.895*
*Body mass index*	*0.045*	*0.652*
*Platelet count*	*0.326*	*0.001*

Abbreviations: HBA1c glycosylated hemoglobin.

### Duration of radiotherapy was significantly associated with CCA IMT

**Figure 1:**
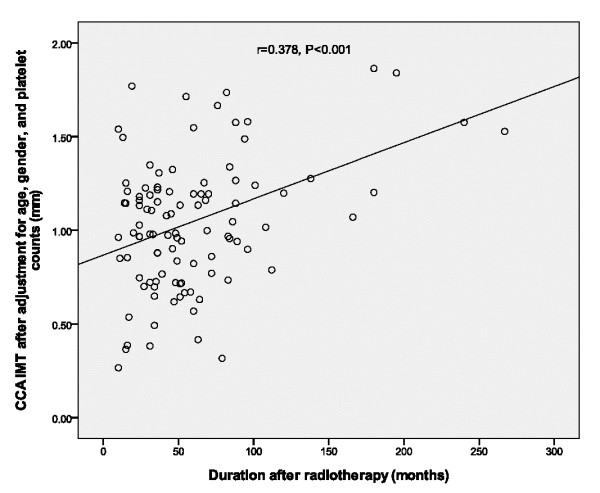
Relationship between common carotid artery (CCA) intima media thickness (IMT) after adjustment for age, gender, and platelet counts, and the duration after radiotherapy in 105 NPC patients.

The two series statistical analyses were subsequently carried out to decipher the relationships between the augmented CCA IMT in NPC patients and duration after radiotherapy or other identified risk variables. Based on correlation analysis, duration after radiotherapy, age, serum levels of hs-CRP and HbA1c, and platelet count significantly correlated with mean CCA IMT (Table 3). Using multiple linear regression analysis in a second series to identify the crucial determinant underlying the augmented CCA IMT in NPC patients, duration after radiotherapy, age, and platelet counts were significantly associated with mean CCA IMT whereas gender, levels of hs-CRP and HbA1c were not (Table 4). Based on the second multiple linear regression analysis that included only those four variables and the formula: CCA IMT (average) = -0.772 + 0.023 × (age) + 0.005 × (duration after radiotherapy) + 0.001 × (platelet counts). The CCA IMT (average) increased linearly with duration after radiotherapy (months) after adjustments for age, gender, and platelet counts (r = 0.378, *p* < 0.001) (Figure 1).

**Table 4:** Multiple regression analysis of the association of IMT with duration of radiotherapy and other candidate risk factors

	***Model 1***			***Model 2***		
	***Regression coefficients***	***Standard error***	***p value***	***Regression coefficients***	***Standard error***	***p value***
*Constant*	*-0.811*	*0.227*	*0.001*	*-0.772*	*0.218*	*0.001*
*Duration after radiotherapy*	*0.005*	*0.001*	*<0.001*	*0.005*	*0.001*	*<0.001*
*Age*	*0.024*	*0.004*	*<0.001*	*0.023*	*0.004*	*<0.001*
*Platelet count*	*0.001*	*0.001*	*0.026*	*0.001*	*0.001*	*0.026*

## Discussion

To date, there has been only one clinical research that focused on CCA IMT, assessed by B-mode ultrasonography and quantifying software, and allowed for direct demonstration of atherosclerotic changes in the vessel wall of post-radiotherapy NPC patients [5]. The current study confirms the posited hypothesis that radiation-induced vasculopathy is a dynamic and progressive process from late radiation effects and that the duration after radiotherapy correlates with the progression of carotid intima-media thickness.

In clinical practice, vascular abnormalities are the predominant histologic changes seen in irradiation for head and neck cancers seen more than 6 months after [4]. Among survivors of the cancer itself, late effects on "bystander" organs like the carotid arteries after irradiation have become increasingly prevalent, with secondary cardio-vascular disease increasing morbidity and mortality. Atherosclerotic and thrombotic complications have drawn the most attention [14-17]. One study published in 2001 showed that the overall prevalence of extra-cranial carotid artery disease was 56 of 71 patients (78.9%). The common and internal carotid arteries were most commonly involved (77.5%), followed by the external carotid (45%) and vertebral (7%) arteries in the post-irradiation group. In contrast, the control group showed a 21.6% involvement of the common and internal carotid arteries, 2.0% external carotid arteries, and no involvement of the vertebral artery [7].

Another study evaluated 910 patients who survived at least five years after irradiation of head and neck tumors had stroke in 6% and clinically significant carotid stenosis in 17% [9]. Furthermore, atherosclerotic plaques result from the interaction between modified lipids, extracellular matrix, monocyte-derived macrophages, and activated vascular smooth muscle cells that accumulate in the arterial wall. Plaque formation is often complicated by conversion into an acute stage of vessel occlusion or thrombo-embolism evolving from the plaque and resulting in acute clinical complications of myocardial infarction and stroke.

The present study, which examined the IMT of CCA in NPC patients post-radiotherapy and on follow-up for more than one year, has three major findings. First, the IMT of CCA is significantly increased in NPC patients compared to healthy control.significantly increased in NPC patients compared to healthy control. Second, 36.2% of NPC cases have carotid plaques and significant risk factors for carotid plaques include age, duration after radiotherapy, and HbA1c levels. The cut-off value of age and duration after radiotherapy for the presence of plaque are 52.5 years and 42.5 months, respectively. Third, age, duration after radiotherapy, *hs*-CRP, HbA1c, and platelet count are positive correlated with IMT. Multiple linear regression analysis reveals that age, duration after radiotherapy and platelet counts are independently associated with CCA IMT. After adjustments for age, gender, and platelet counts, IMT increases in a linear manner with the duration after radiotherapy.

Although this study demonstrates that the duration after radiotherapy correlates with the progression of carotid intima-media thickness, there are two main limitations. First, the study does not exclude patients with underlying diseases of DM and hyperlipidemia and expressions of hs-CRP level and the platelet activation markers and IMT of CCA that may be influence by drugs (e.g. statins and calcium channel blockers), which may cause potential bias in the statistical analysis. Second, this is a cross-sectional study and therefore subject to bias of unmeasured factors. It is also not possible to assess the effect of radiotherapy on the change of IMT in the same individual.

In conclusion, radiation-induced vasculopathy is a dynamic and progressive process from the late radiation effects. This is important in the understanding of the pathogenesis of atherosclerosis in NPC patients after radiotherapy. Extra-cranial color-coded duplex sonography may be added in routine follow-up studies of NPC patients aged ≥50 years old at 40 months post-radiotherapy to reduce morbidity and mortality from atherosclerosis-related diseases. Large-scale trials evaluating the relationship between change of IMT and effects of radiotherapy in NPC patients are warranted to clarify optimal strategy.

### Ethics approval

The study was approved by Chang Gung Memorial Hospital’s Institutional Review Committee on Human Research.

## Competing interests

The authors indicated no potential conflicts of interests.

## Authors’ contributions

Conception and design: Cheng-Hsien Lu; Provision of study materials or patients: Cheng-Hsien Lu; Collection and assembly of data: Tai-Lin Huang, Hsuan-Chih Hsu, Hui-Chun Chen, Hsin-Ching Lin, Chih-Yen Chien Fu-Min Fang, Chih-Cheng Huang, Hsueh-Wen Chang, Wen-Neng Chang, Chi-Ren Huang, Nai-Wen Tsai, Chia-Te Kung, Hung-Chen Wang, Wei-Che Lin, Yu-Jun Lin, Ben-Chung Cheng, Yu-Jih Su, Ya-Ting Chang; Data analysis and interpretation: Chuang-Rung Chang, Teng-Yeow Tan and Cheng-Hsien Lu; Manuscript writing: All authors; Final approval of manuscript: All authors.
